# Supplementary figures and images for: NormiRazor: tool applying GPU-accelerated computing for determination of internal references in microRNA transcription studies
Source: BMC Bioinformatics. 2020 Sep 29;21:425. doi: 10.1186/s12859-020-03743-8 (PMC7523363; doi:10.1186/s12859-020-03743-8)

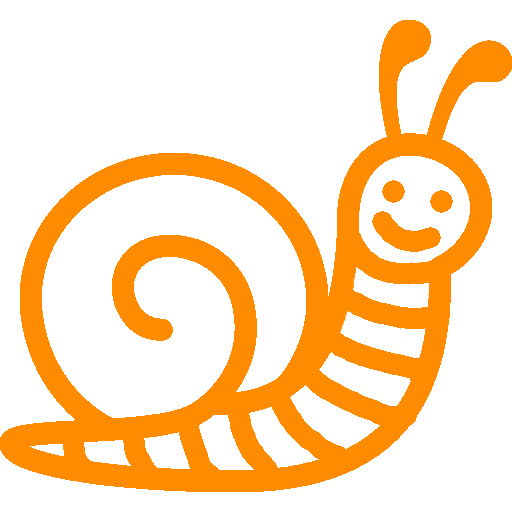

Supplement: Supplementary file 3 — Additional file 3 Archive with source code. This archive contains NormiRazor source code. [file 12859_2020_3743_MOESM3_ESM.zip › normirazor-master/favicon.png]

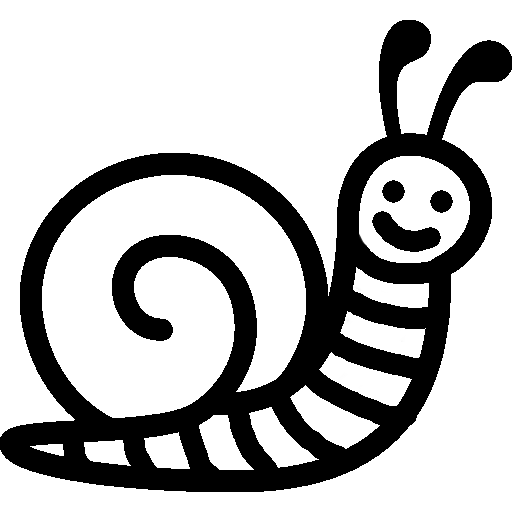

Supplement: Supplementary file 3 — Additional file 3 Archive with source code. This archive contains NormiRazor source code. [file 12859_2020_3743_MOESM3_ESM.zip › normirazor-master/norm-logo.png]
